# Supplementary material for: Fabrication and Characterization of a Self-Powered n-Bi2Se3/p-Si Nanowire Bulk Heterojunction Broadband Photodetector
Source: Nanomaterials (Basel). 2022 May 26;12(11):1824. doi: 10.3390/nano12111824 (PMC9182573; doi:10.3390/nano12111824)
Supplement: Supplementary file 1 [file nanomaterials-12-01824-s001.zip › nanomaterials-1699853-supplementary.pdf]

nanomaterials

Supplementary Material

Fabrication and Characterization of a Self-Powered n-Bi_2Se_3/p-Si Nanowire Bulk Heterojunction Broadband Photodetector

Xuan Wang [1], Yehua Tang [1], Wanping Wang [1], Hao Zhao [2], Yanling Song [1], Chaoyang Kang [1,*] and KeFan Wang [1,*]

1 Henan Province Key Laboratory of Photovoltaic Materials, Henan University, Kaifeng 475004, China; 104754190988@henu.edu.cn (X.W.); 80020012@vip.henu.edu.cn (Y.T.); 104754191019@henu.edu.cn (W.W.); songyl@163.com (Y.S.)

2 School of Physics and Electronics, Henan University, Kaifeng 475004, China; 18211973341@163.com

* Correspondence: kangcy@vip.henu.edu.cn (C.K.); kfwang@henu.edu.cn (K.-F.W.)

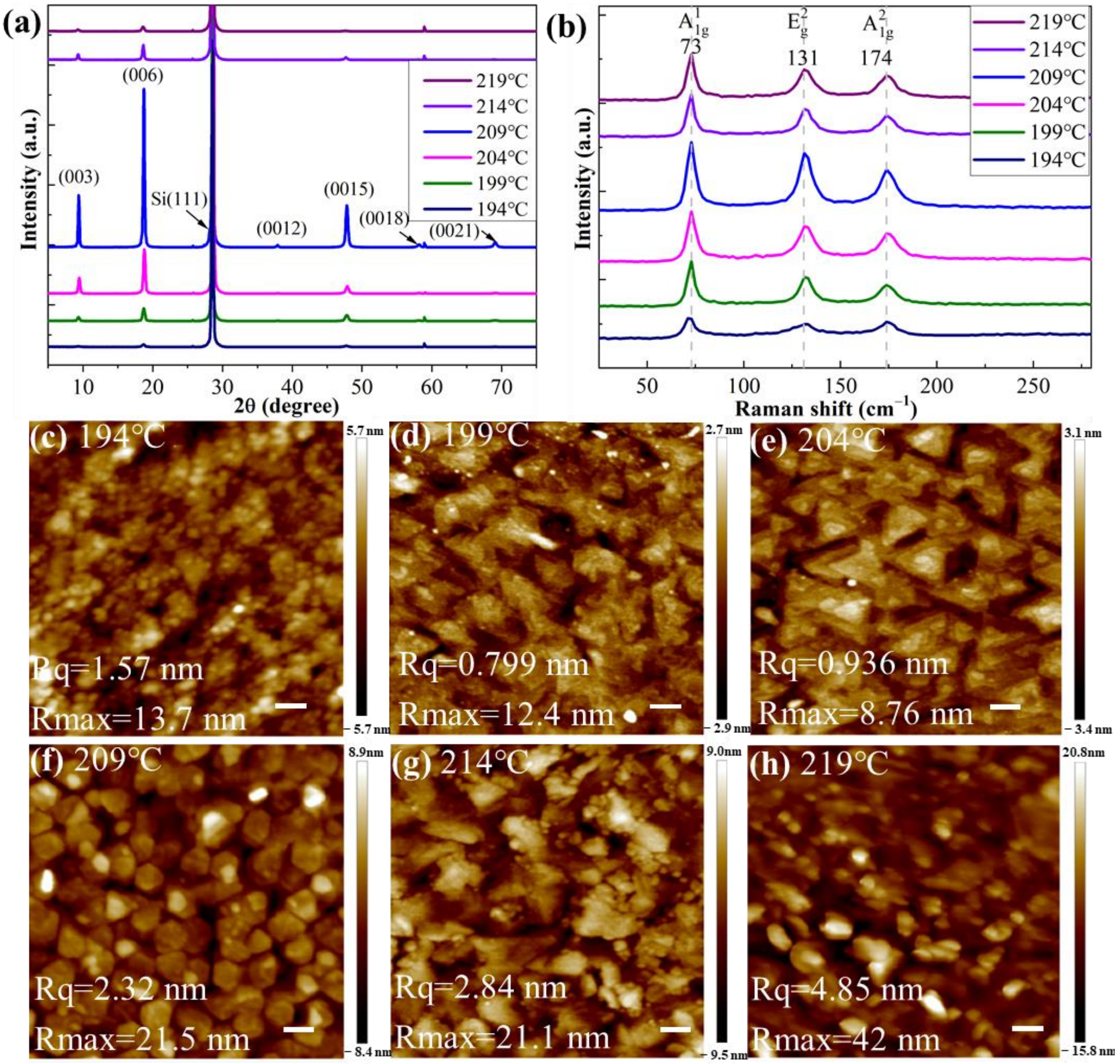

Figure S1. Characterization of Bi_2Se_3 films grown at the different Se cell temperatures (194℃~219℃) and at the same Bi cell temperature of 750℃ and the growth temperature of 320℃: (**a**) XRD; (**b**) Raman; (**c**)-(**h**) AFM of sample surface. Scale bars in (c)-(h) are all 100 nm.

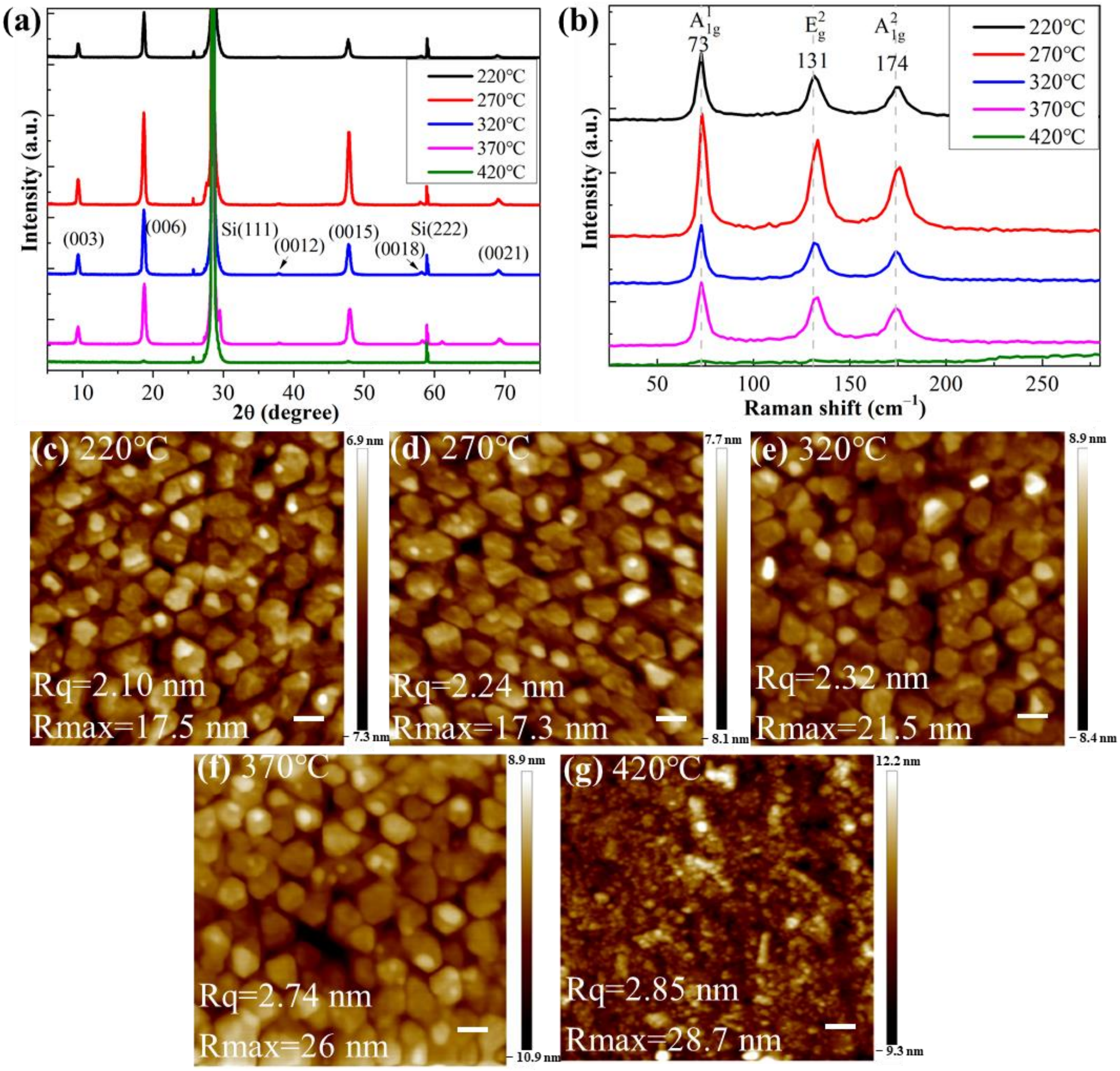

Figure S2. Characterization of Bi_2Se_3 films grown at the different growth temperatures (220℃~420℃) and at the same Bi cell temperature of 750℃ and Se cell temperature of 209℃: (a) XRD; (b) Raman; (c)-(g) AFM of sample surface. Scale bars in (c)-(g) are all 100 nm.

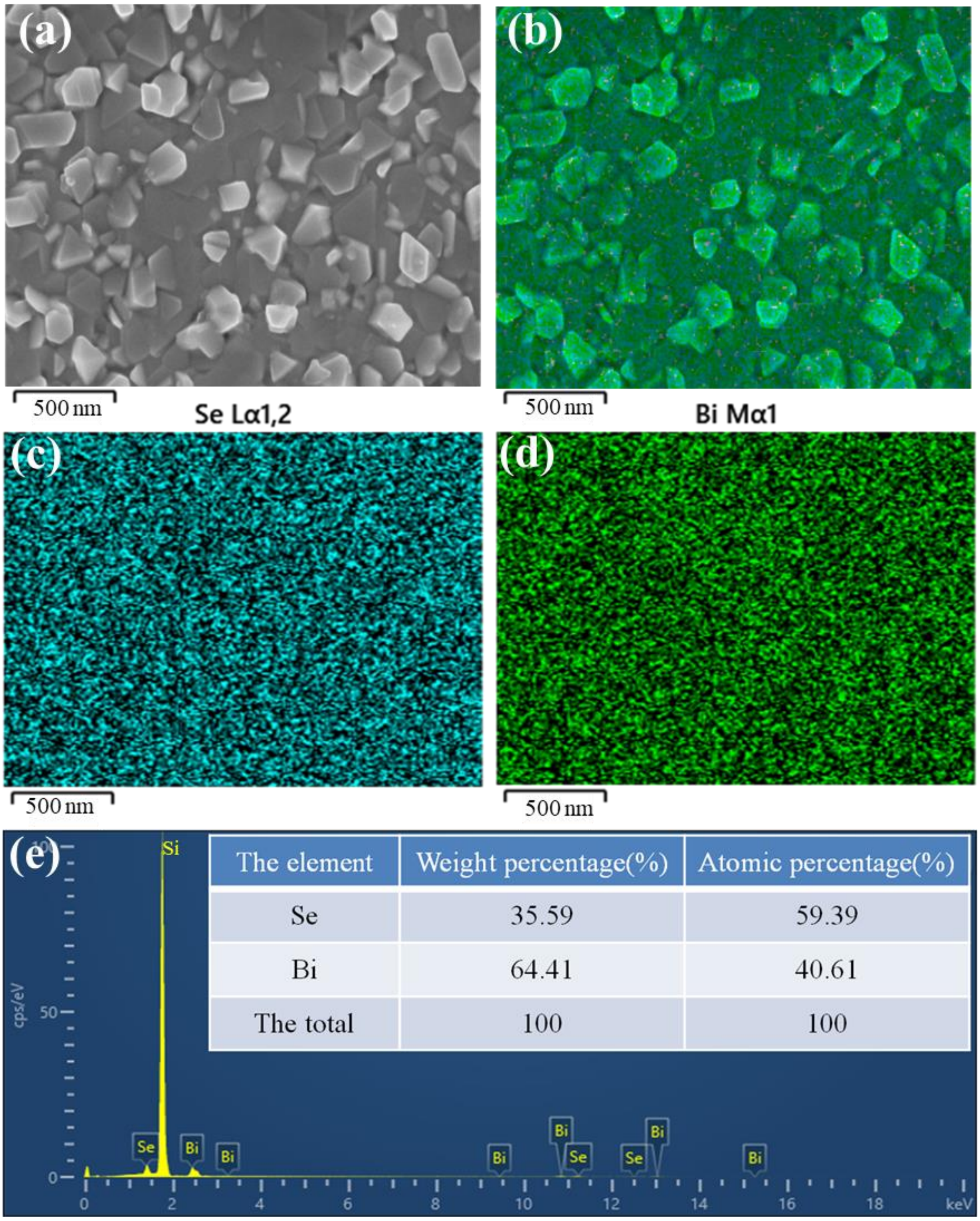

Figure S3. (**a**) Surface SEM image of Bi_2Se_3 thin films; (**b**) EDS stratification diagram of Bi_2Se_3 thin film; (**c**) Se atomic distribution map; (**d**) Bi atomic distribution map; (**e**) EDS spectrum of Bi_2Se_3 thin film.

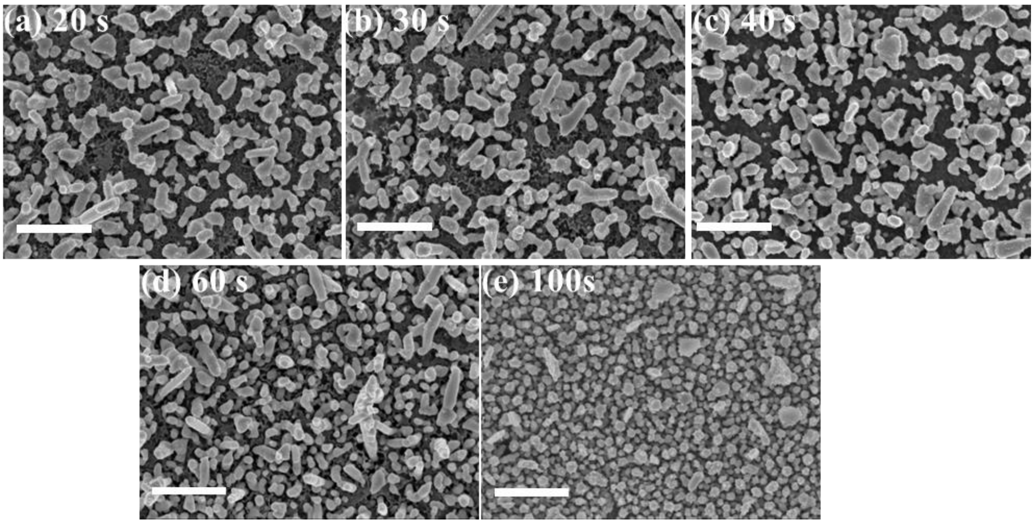

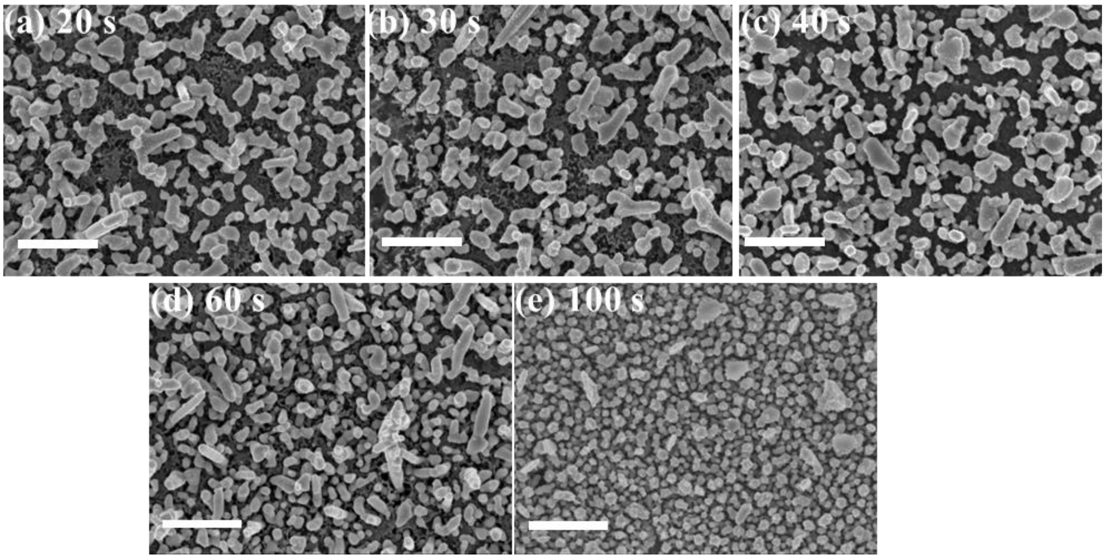

Figure S4. Surface SEM images of Ag NPs with different deposition times: (**a**) 20 s; (**b**) 30 s; (**c**) 40 s; (**d**) 60 s; (**e**) 100 s. Scale bars in (a)-(e) are all 1 μm.

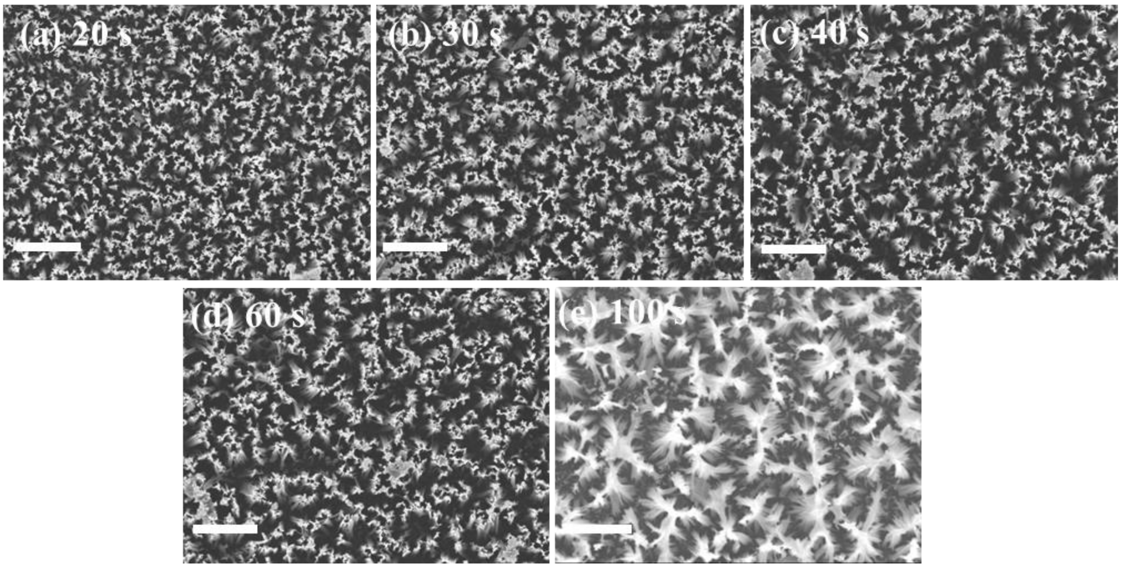

Figure S5. Surface SEM images of the Si NWs with the same etching time of 10 min and the different deposition times of Ag NPs: (**a**) 20 s; (**b**) 30 s; (**c**) 40 s; (**d**) 60 s; (**e**) 100 s. Scale bars in (a)-(e) are all 2 μm.

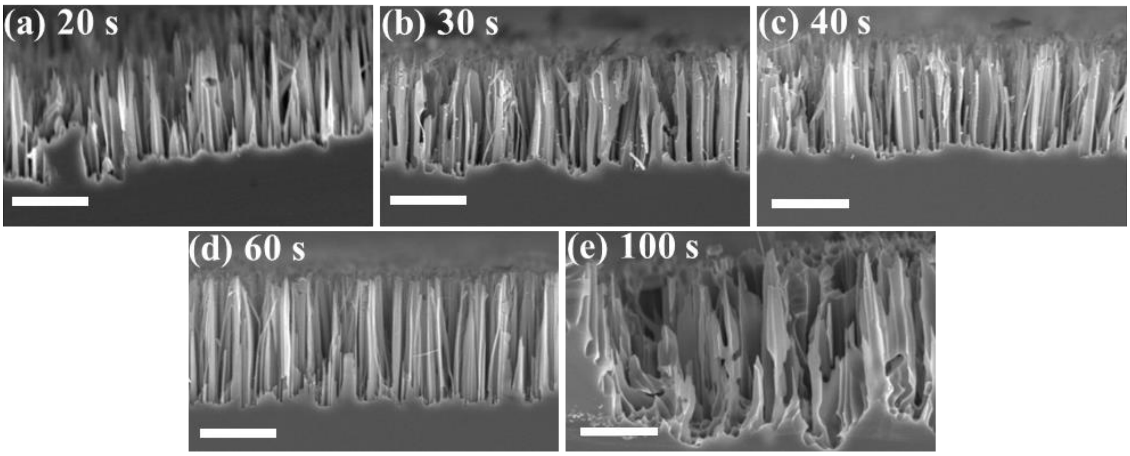

Figure S6. Cross-sections SEM images of Si NPs with the same etching time of 10 min and the different deposition times of Ag NPs: (**a**) 20 s; (**b**) 30 s; (**c**) 40 s; (**d**) 60 s; (**e**) 100 s. Scale bars in (a)-(e) are all 2 μm.

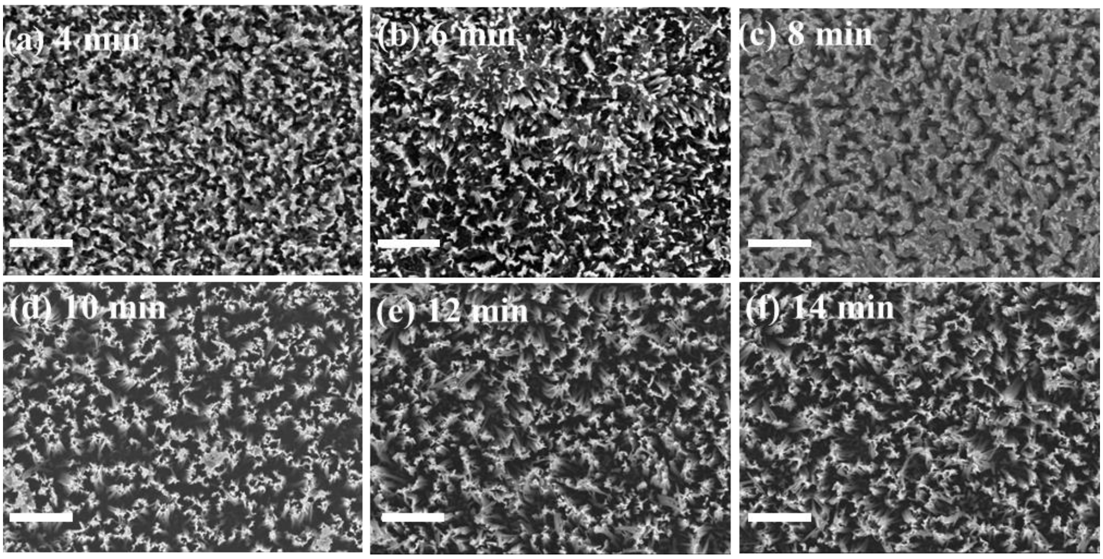

Figure S7. Surface SEM images of Si NWs with the same Ag NPs deposition time of 60 s and the different etching time: (**a**) 4 min; (**b**) 6 min; (**c**) 8 min; (**d**) 10 min; (**e**) 12 min; (**f**) 14 min. Scale bars in (a)-(f) are all 2 μm.

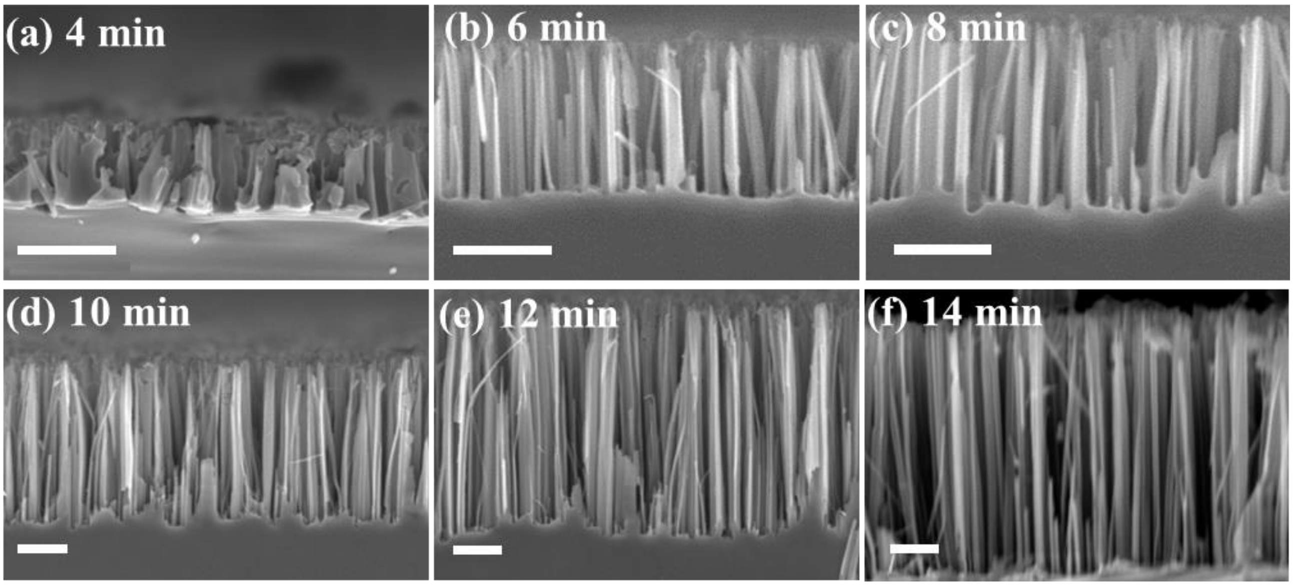

Figure S8. Cross-section SEM images of Si NWs with the same Ag NPs deposition time of 60 s and the different etching time: (**a**) 4 min; (**b**) 6 min; (**c**) 8 min; (**d**) 10 min; (**e**) 12 min; (**f**) 14 min. Scale bars in (a)-(f) are all 1 μm.

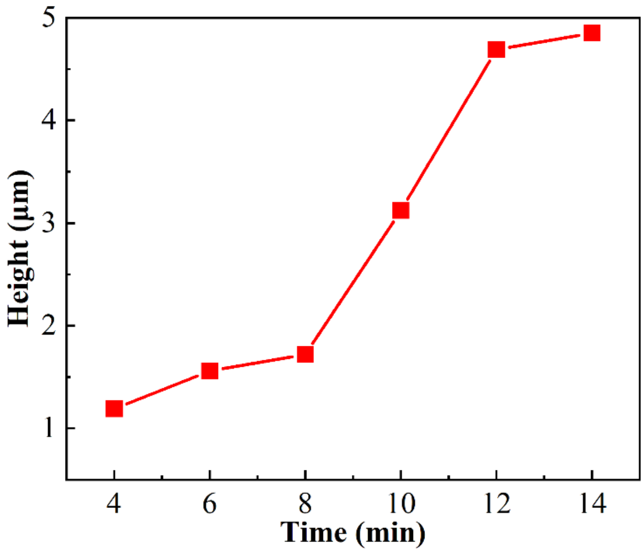

Figure S9. The height of Si NWs as a function of the etching time.

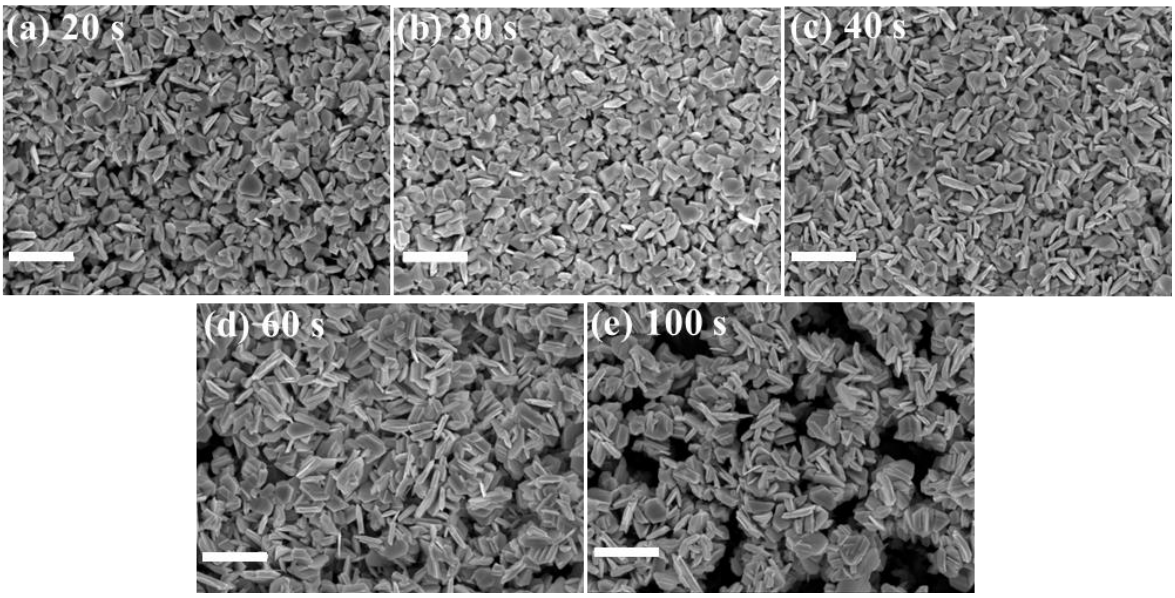

Figure S10. Surface SEM images of Bi_2Se_3/Si NWs samples with the same etch time of 10 min and the different deposition time of Ag NPs: (**a**) 20 s; (**b**) 30 s; (**c**) 40 s; (**d**) 60 s; (**e**) 100 s. Scale bars in (a)-(e) are all 1 μm.

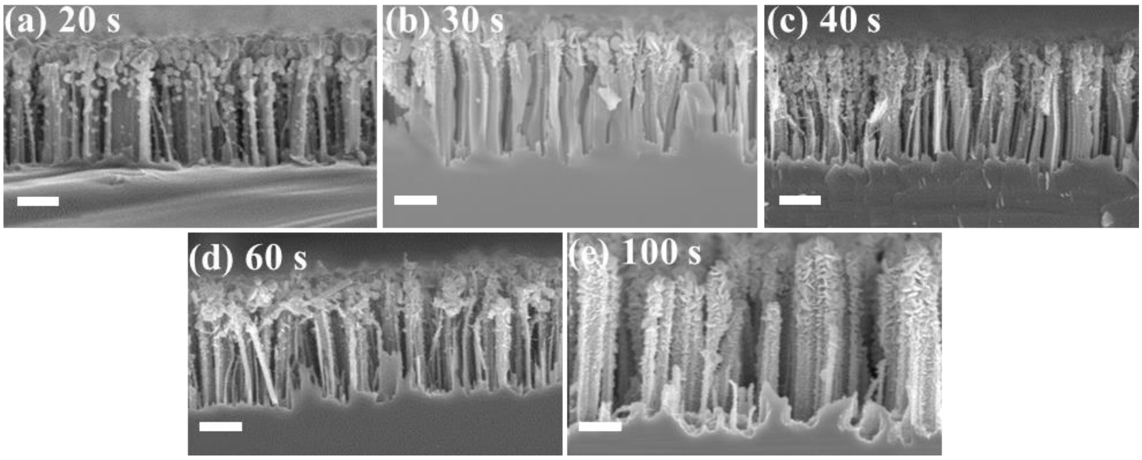

Figure S11. Cross-section SEM images of Bi_2Se_3/Si NWs samples with the same etch time of 10 min and the different deposition times of Ag NPs: (**a**) 20 s; (**b**) 30 s; (**c**) 40 s; (**d**) 60 s; (**e**) 100 s. Scale bars in (a)-(e) are all 1 μm.

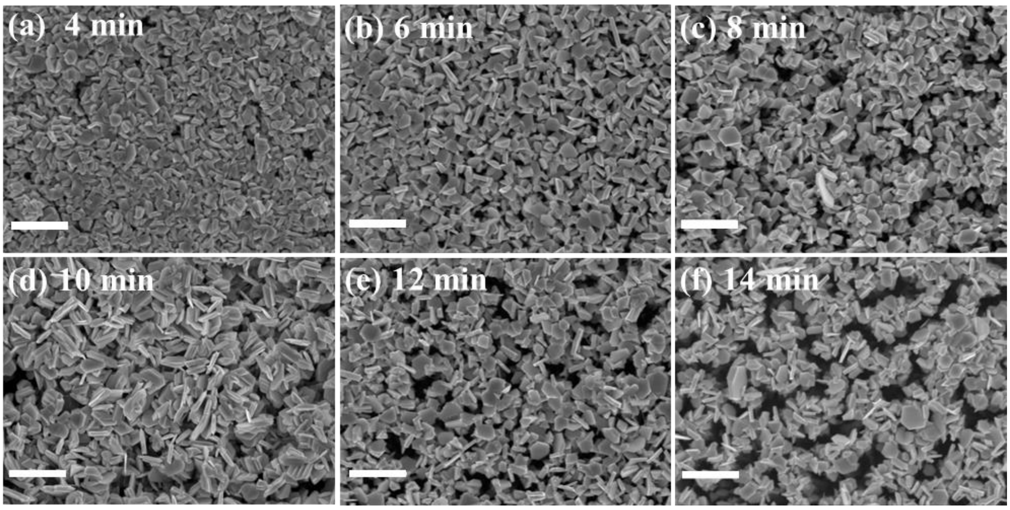

Figure S12. Surface SEM images of Bi_2Se_3/Si NWs samples with the same deposition times (60 s) of Ag NPs and the different etching times: (**a**) 4 min; (**b**) 6 min; (**c**) 8 min; (**d**) 10 min; (**e**) 12 min; (**f**) 14 min. Scale bars in (a)-(f) are all 1 μm.

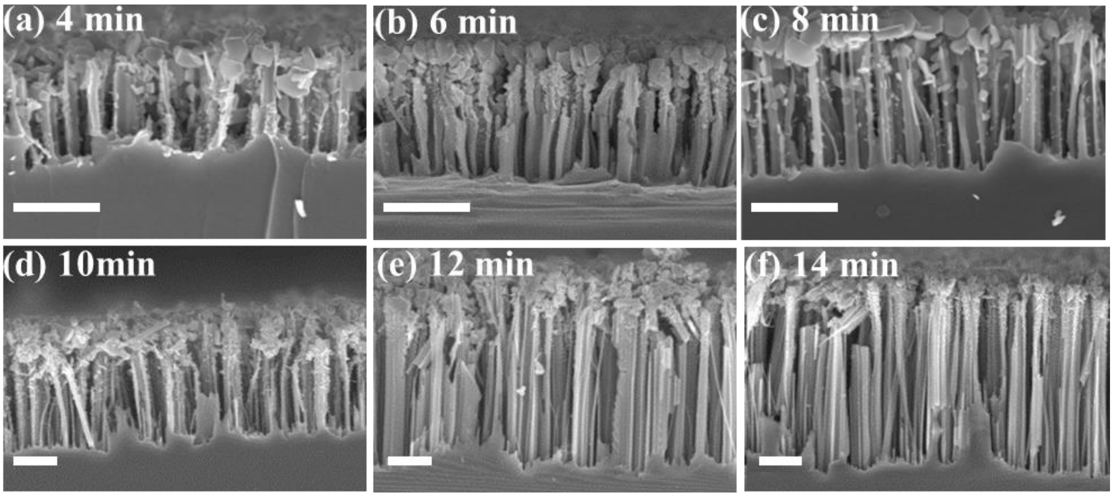

Figure S13. Cross-section SEM images of Bi_2Se_3/Si NWs samples with the same deposition times (60s) of Ag NPs and the different etching times: (**a**) 4 min; (**b**) 6 min; (**c**) 8 min; (**d**) 10 min; (**e**) 12 min; (**f**) 14 min. Scale bars in (a)-(f) are all 1 μm.

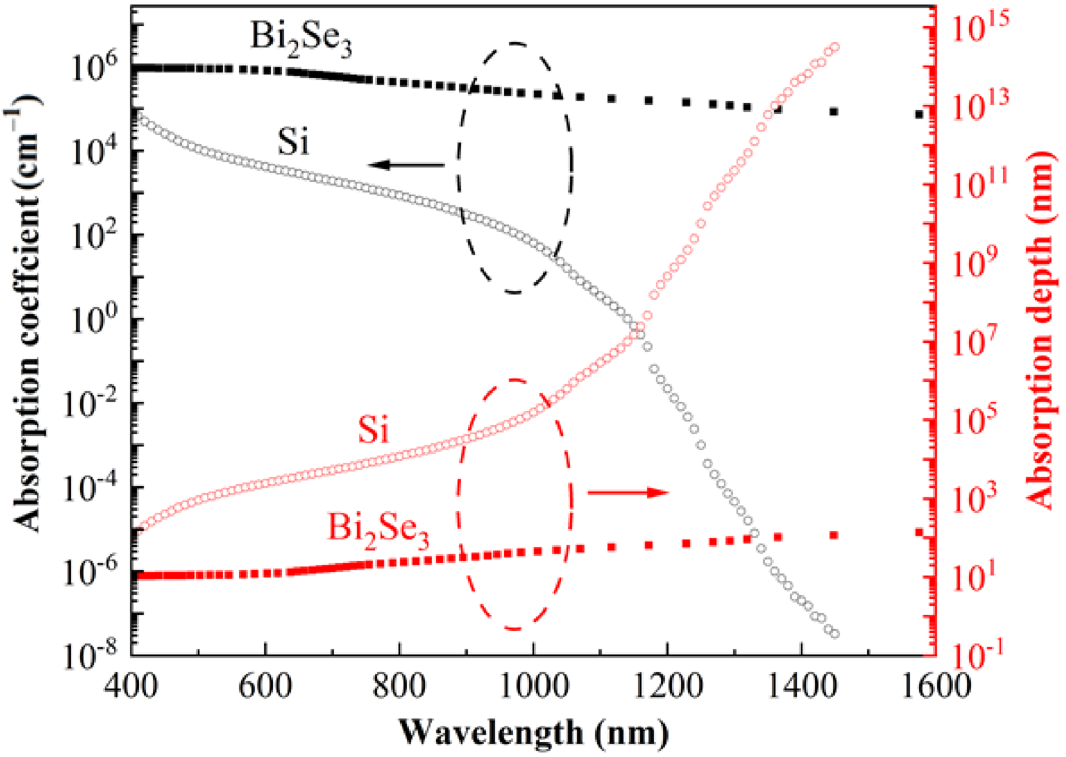

Figure S14. Absorption coefficient and absorption depth of silicon and Bi_2Se_3[24, 25].

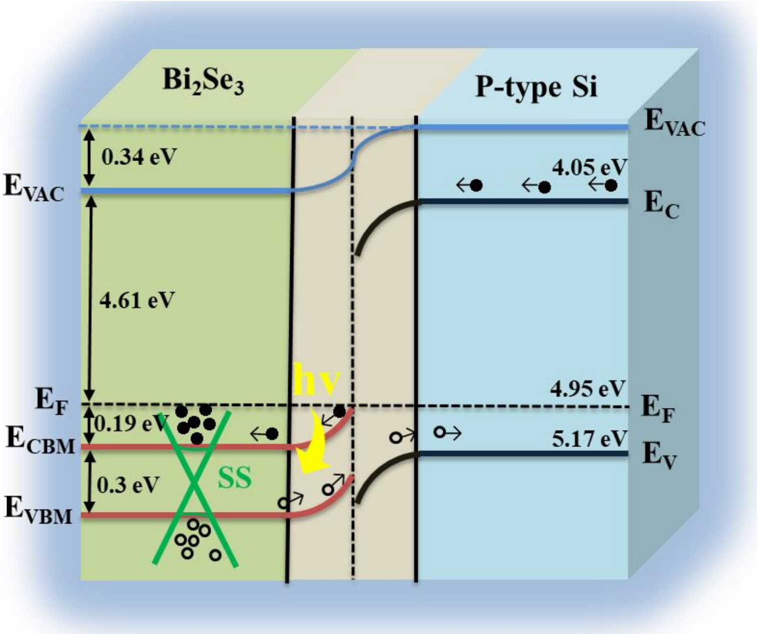

Figure S15. Energy-band diagrams for a n-Bi_2Se_3/p-Si NWs heterostructure photodetector. Evac is the vacuum energy level. E_{CBM} and E_{VBM} are the conduction-band bottom and valence-band top of the Bi_2Se_3 film, respectively. E_C and E_V denote the conduction-band bottom and valence-band top of Si, respectively, and E_F is the Fermi level. The solid and hollow spots represent photogenerated electrons and holes, respectively.

Table S1. Performance comparison of our Bi_2Se_3/Si NWs photodetector with previous Bi_2Se_3/Si photodetectors.

Sample	Wavelength (nm)	Responsivity	D*(Jones)	I_p/I_d	τ_r/τ_f (ms)	References

Bi_2Se_3/p-Si NWs	390-1700	14.17 mA/W (960 nm) 84.3 mA/W (390 nm) 7 mA/W (1.7 μm)	1.79×10^{9} (960 nm) 2.38×10^{10} (390 nm) 8.28×10^{8} (1.7 μm)	61.7 (980 nm)	3/1 (980 nm)	This work
Bi_2Se_3 Nanoflakes/p-Si NWs	300-1000	938.4 A/W (900 nm)	2.30×10^{13} (900 nm)	2.5 (900 nm)	41/79 (900 nm)	[21]
Vertical Cu-dope Bi_2Se_3 nanoplate/p-Si	400-1200	168.9 mA/W (900 nm)	1.85×10^{11} (900 nm)	113 (900 nm)	4/4 (900 nm)	[19]
Bi_2Se_3/pyramidal n-Si	635-2700	3.06×10^{-5} mA/W (1.5 μm) 1.8×10^{-5} mA/W (2.7 μm)	1.37×10^{5} (1.5 μm) 1.53×10^{6} (2.7 μm)	1.01×10^{4} (1.5 μm) 1.42 (2.7μm)	0.52/0.44 (1.55 μm) 0.585/0.535 (2.7μm)	[17]
Bi_2Se_3 NWs/n-Si	380-1310	924.2 A/W (808 nm)	2.38×10^{12} (808 nm)	10 (808 nm)	45/47 (808 nm)	[18]
Bi_2Se_3/n-Si	350-1100	24.28 A/W (808 nm)	4.39×10^{12} (808 nm)	1.55×10^{5}(808 nm)	2.5/5.5 $\times10^{-3}$ (808 nm)	[16]
**MoS_2**/Si	450-1050	300 mA/W	10^{23} (808 nm)	--	3/40 $\times10^{-3}$ (808 nm)	[51]
Reduced Graphene Oxide (RGO)/Si NWs	532-10,600	9 mA/W (10.6 μm)	--	--	70 (10.6 μm)	[52]
